# Biological Load and Tray Position Shape Larval Yield and Frass Production in a Stacked *Hermetia illucens* Rearing System

**DOI:** 10.3390/ani16182900

**Published:** 2026-09-15

**Authors:** Alondra Guerrero-Hernández, Aurelio Guevara-Escobar, Juan Fernando García-Trejo, Ximena Galilea Ruiz-Maldonado, Ana Angelica Feregrino-Pérez

**Affiliations:** 1 Facultad de Ciencias Naturales, Universidad Autónoma de Querétaro, Juriquilla, Querétaro 76230, Mexico; aguerrero70@alumnos.uaq.mx (A.G.-H.); xruiz08@alumnos.uaq.mx (X.G.R.-M.); 2 Facultad de Ingeniería, Universidad Autónoma de Querétaro, Amazcala, Querétaro 76265, Mexico; fernando.garcia@uaq.mx (J.F.G.-T.); geli@uaq.mx (A.A.F.-P.)

**Keywords:** black soldier fly, BSFL, initial larval mass, rearing trays, stacked systems, frass, substrate moisture

## Abstract

Industrial black soldier fly (*Hermetia illucens*) production commonly relies on stacked tray systems, yet little is known about how tray position and biological load influence production heterogeneity. This study evaluated whether different initial biological loads, imposed by varying the initial larval mass while maintaining a constant numerical density, together with tray position, influence larval biomass and dry frass production. Same-age third-instar larvae were seeded at a constant density of 9000 larvae per tray under five biological-load treatments (29, 54, 64, 97 and 200 g tray^−1^, fresh weight basis). Initial biological load affected wet larval biomass, dry larval biomass, and dry frass production. However, higher initial biological load did not produce proportional gains in final biomass. The lowest initial biological load resulted in the greatest final wet larval biomass (3.54 kg tray^−1^) whereas the highest initial biological load resulted in only 1.75 kg tray^−1^ (p=0.025). Intermediate tray positions produced significantly greater wet larval biomass than the bottom tray. Mean substrate temperature did not differ among tray positions, whereas moisture declined over time and varied by position. These findings demonstrate that production consistency in stacked *H. illucens* rearing systems depends not only on biological load but also on tray-level microenvironmental heterogeneity, providing practical guidance for the design and management of industrial stacked-rearing systems.

## Simple Summary

Black soldier fly larvae (*Hermetia illucens*) are increasingly used to convert organic materials into valuable insect biomass and frass. However, production can vary considerably among trays in stacked rearing systems. This study evaluated whether initial biological load and vertical tray position contribute to this variability under pilot-scale rearing conditions. Forty trays were stocked with 9000 larvae each using five initial larval biomass levels and arranged at five vertical positions within stacks. Initial biological load strongly influenced production, but increasing the starting larval biomass did not result in greater final yield. The lowest initial load produced the greatest wet larval biomass, whereas the highest load produced substantially less. Tray position also affected production, with intermediate positions generally yielding more larval biomass than the lowest position. Moisture conditions varied among trays, whereas temperature differences among positions were limited. These results show that both biological load and the spatial arrangement of trays can create important production heterogeneity and should be considered when designing and managing stacked black soldier fly rearing systems.

## 1. Introduction

Organic residues remain a major management challenge because many municipal, agro-industrial, and livestock-derived waste streams still decompose under poorly controlled conditions. This pathway promotes odors, leachate formation, methane emissions, and the proliferation of nuisance organisms, while also losing nutrients that could be recovered for productive uses [1,2,3,4]. Biological conversion systems can redirect part of this organic matter into circular value chains. Among these systems, black soldier fly larvae (BSFL), *Hermetia illucens* (L.) (Diptera: Stratiomyidae), have attracted particular attention because they rapidly consume heterogeneous organic substrates and convert them into larval biomass and a residual material commonly referred to as frass [5,6,7,8]. Larval biomass can be processed as an ingredient for animal feed or other bioproducts, whereas the residual fraction commonly referred to as frass may contain larval excreta, residual or partially consumed substrate, shed exuviae, and other organic material generated during rearing. This material may be recovered as an organic fertilizer or soil amendment when it meets safety and quality requirements [9,10,11].

The production value of a BSFL system depends not only on waste reduction, but also on the predictable recovery of harvestable larval biomass. In practice, this predictability is difficult to achieve because larval growth integrates the effects of substrate composition, moisture, temperature, feeding rate, larval age at seeding, and rearing scale [12,13,14,15,16]. Moisture controls feed accessibility, larval mobility, and microbial activity. Low moisture can restrict ingestion and slow development, whereas excessive moisture can reduce aeration, promote leachate formation, and increase mortality risk [14,17,18]. Temperature also regulates consumption rate and development time; BSFL usually perform well under warm mesophilic conditions, but larval metabolism and microbial fermentation can generate internal heat that differs from ambient air temperature [19,20,21,22]. These processes become especially important when producers increase the amount of substrate or the number of larvae per production unit.

In this study, biological load refers to the total fresh biomass of larvae introduced into a rearing unit at the beginning of the grow-out period. Unlike numerical larval density, which describes the number of individuals per unit area or rearing unit, biological load incorporates differences in individual larval mass and therefore represents the initial amount of metabolically active larval biomass placed in each tray.

Seeding density is one of the most direct management variables in BSFL production. It determines the number of larvae competing for feed, the rate at which the substrate is processed, the amount of metabolic heat generated, and the uniformity of individual growth. Early optimization work showed that larval density and feeding rate jointly shape vegetable-waste bioconversion, with density acting as a major driver of process performance [23]. Subsequent experiments confirmed that density interacts with dietary nutrient concentration and can affect larval performance, body composition, and the balance between individual larval size and total biomass recovery [24]. More recent work has emphasized that density should not be interpreted as an isolated parameter: its effect depends on moisture, feed availability, substrate physical structure, aeration, and rearing scale [25,26,27]. Consequently, a density that maximizes individual larval weight may not maximize total tray productivity, and a density that performs well in small containers may fail when transferred to larger or deeper production units.

Scale-up introduces another source of variation: the physical arrangement of rearing units. Industrial and semi-industrial BSFL systems commonly use plastic boxes, trays, or modular reactors placed on racks to increase production per unit of floor area. This stacked configuration improves space use, but it can also create microenvironmental gradients. Tray position may affect heat exposure, air exchange, evaporation, and the accumulation or loss of moisture, especially when heating elements or warm air sources are placed above, below, or along one side of the stack. Because larvae and associated microbes generate heat while consuming the substrate, each tray can behave as a small bioreactor rather than as a passive container. Larger rearing scales can reach higher internal temperatures than smaller scales even when the nominal feed allowance per larva is maintained [25]. These observations suggest that vertical position within a stack may modify larval yield, frass recovery, and the consistency of production among boxes.

Despite the operational relevance of stacked rearing systems, most BSFL studies still report density, feeding rate, diet, or moisture under conditions that do not explicitly test the position of cultivation boxes within a vertical pile or rack. This limits the translation of experimental results to production rooms where trays are routinely stacked and where environmental control is applied at the room or rack level rather than at the individual-box level. The issue is particularly important when external heaters are located in a fixed position relative to the trays, because the upper, middle, and lower boxes may experience different thermal and moisture conditions during the same production cycle. Without this information, producers may attribute differences in larval biomass to biological variability or substrate heterogeneity when part of the variation may arise from the physical arrangement of the rearing system.

This study addresses that gap by evaluating the variation in *H. illucens* larval yield as a function of initial biological load and tray arrangement in stacked cultivation boxes. We focus on two practical questions: first, how does initial biological load affect the recovery of larval biomass and frass under the same production protocol; and second, does the position of the box within a stack contribute to systematic differences in production? We hypothesized that larval yield would respond nonlinearly to initial biological load and that tray position would modify production because stacked boxes experience different microenvironmental conditions. The results are intended to support operational recommendations for selecting biological load and defining the maximum number or arrangement of boxes that can be stacked without compromising productive consistency.

## 2. Materials and Methods

### 2.1. Study Site and Rearing Facility

**Figure 1:**
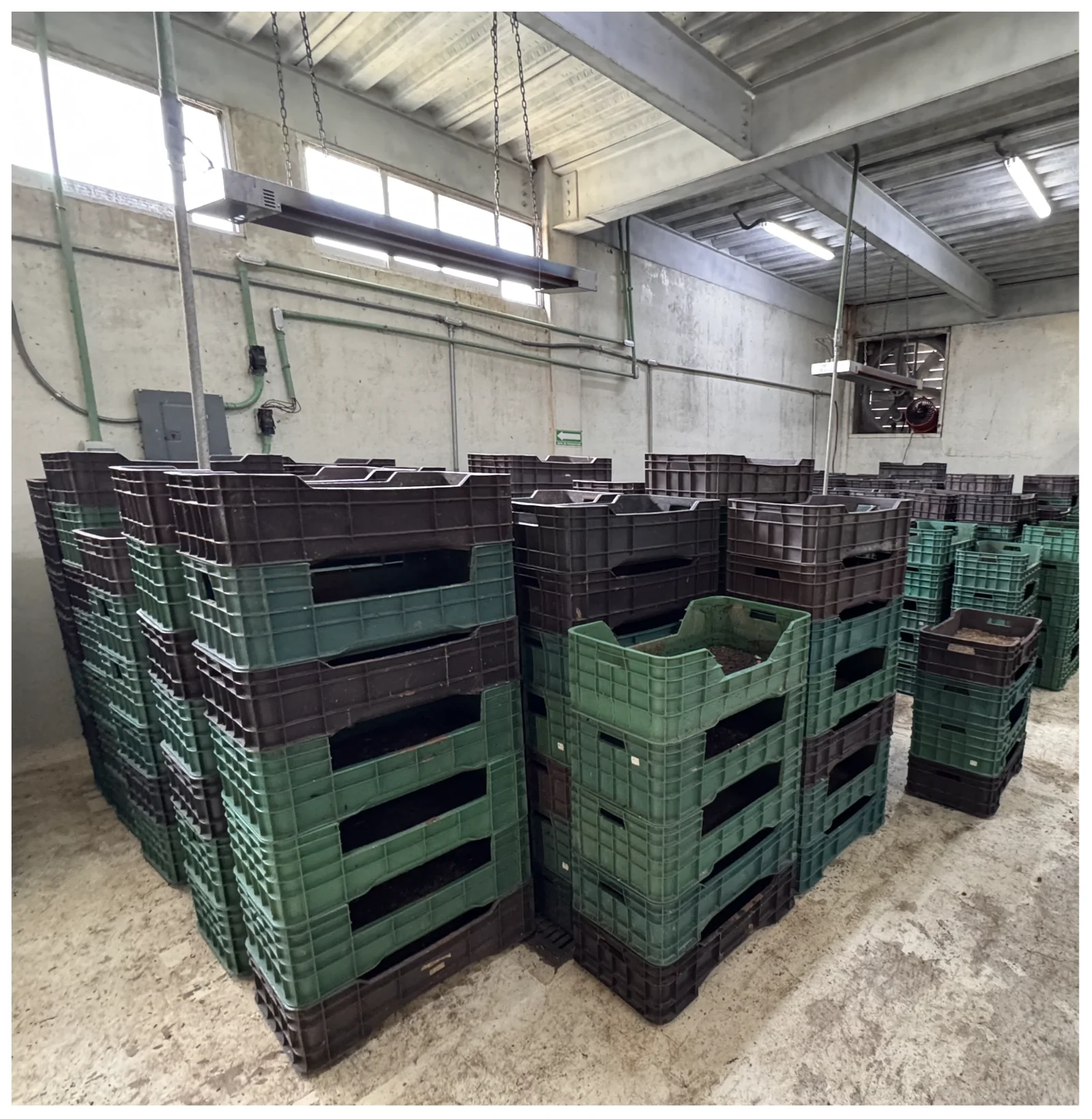
General arrangement of the pilot-scale *Hermetia illucens* rearing facility at the Amazcala Campus, showing the vertical tray stacks, hanging heating equipment used for environmental control, and the surrounding rearing space.

The study was conducted at the pilot production facility for *Hermetia illucens* located at the Amazcala Campus of the School of Engineering, Universidad Autónoma de Querétaro, El Marqués, Querétaro, Mexico (20°42′21.93″ N, 100°15′35.06″ W; 1921 m a.s.l.). The rearing area covered approximately 300 m^2^. The facility remained closed at night and was operated with cross-ventilation during the day. Larvae were reared under the natural day–night light cycle of the facility; fluorescent lights installed in the rearing room were used only for routine facility operations and were not used to establish, extend, or otherwise control the photoperiod. Relative humidity of the room air was not recorded during the experiment. The general arrangement of the stacked trays, heating equipment, and rearing area is shown in Figure 1.

**Figure 2:**
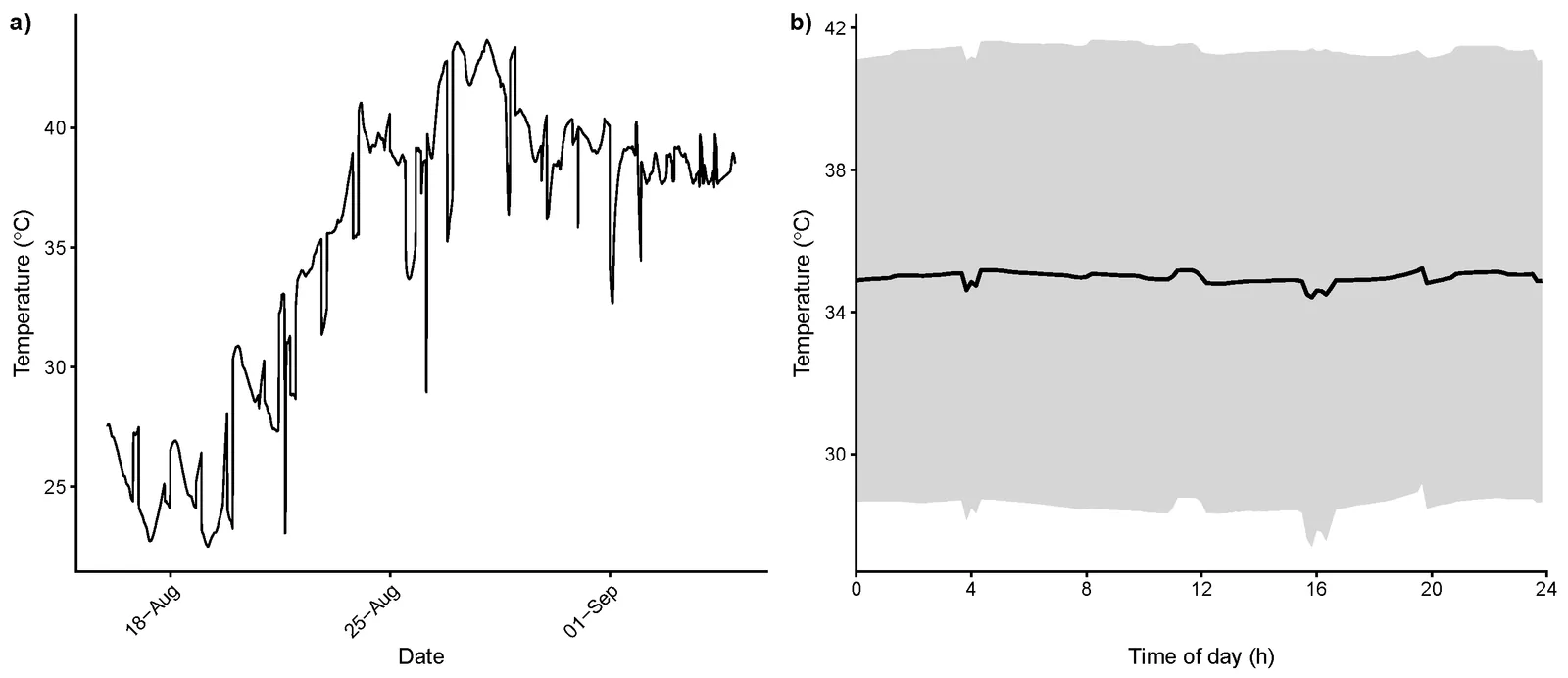
Ambient temperature in the BSFL rearing facility, measured at 20-s intervals in the space between the experimental tray stacks. (**a**) Temperature variation throughout the study period, showing the overall temporal trend. (**b**) Mean diurnal temperature pattern, with the shaded area representing ±1 standard deviation among days.

Lateral domestic-type fans and custom-designed overhead electric heaters were used to maintain environmental conditions suitable for larval growth. Therefore, airflow was used as part of the operational management of the rearing area, but it was not calibrated or quantified as an experimental factor. This condition should be considered when interpreting tray-position effects, because domestic fans may generate heterogeneous air movement around stacked trays rather than a uniform vertical airflow. Mean ambient temperature during the study period was 37.8 °C, ranging from 21.0 to 43.7 °C. Temperature showed an overall increasing trend over the course of the study, together with a relatively stable diurnal pattern (Figure 2).

### 2.2. Larval Material and Experimental Treatments

Seven-day-old third-instar larvae were screened using progressive sieves to obtain groups that differed in larval size. For each size group, five samples of 50 larvae were weighed to estimate the mean individual larval mass. These measurements were used to establish the initial biological load of each treatment (Table 1). Five levels of initial biological load were created by varying the initial larval mass while maintaining a constant numerical density of 9000 larvae per tray. For clarity, treatments are referred to throughout the manuscript by their rounded initial biological loads (29, 54, 64, 97, and 200 g tray^−1^), whereas Table 1 reports the corresponding measured values. Because the availability of same-age larvae differed among size classes, the number of experimental replicates was unbalanced. The experimental unit was a plastic tray measuring 53 cm long, 35 cm wide, and 31 cm high, with a base area of 0.1855 m^2^. A lateral opening of 10 cm × 20 cm was cut into each tray to improve ventilation.

Experimental trays were arranged in two parallel groups facing one another across an aisle approximately 1.2 m wide. Each group consisted of four vertical stacks containing five experimental trays per stack, resulting in eight physical stacks and 40 experimental trays. A 1.75-m-long overhead heater was positioned above the space between the two groups. Each pair of directly opposing stacks, one from each group, was considered a spatial block because both occupied comparable longitudinal positions relative to the heater. Thus, the experimental layout comprised four spatial blocks, each containing two opposing stacks and ten trays.

Treatment allocation was restricted by the availability of same-age larvae within the different larval-size classes. The 29- and 63-g biological-load treatments were randomly allocated among trays within one group of four stacks, whereas the 53-, 96-, and 200-g treatments were randomly allocated within the opposing group. Consequently, replication was unbalanced and the treatments were not completely crossed with physical group, block, or tray position.

Tray position within each stack was evaluated as a second experimental factor, with Position 1 corresponding to the lowest tray and Position 5 to the uppermost tray. Each stack was covered with an additional empty tray. To reduce edge effects, the experimental groups were surrounded by non-experimental rearing stacks arranged in a similar configuration.

Each tray received 6 kg of fresh substrate at each feeding event, resulting in an initial substrate depth of approximately 6 cm. Under these conditions, a constant density of 9000 larvae per tray (48,520 larvae m^−2^) was used throughout the experiment. This density is representative of intensive stacked black soldier fly production systems and enabled all treatments to be evaluated under identical spatial and feeding conditions, so that treatment differences reflected initial biological load rather than differences in feed allocation or available rearing space [28]. Maintaining a constant numerical density while varying the initial biological load allowed the effects of biological load to be separated from those of larval crowding, thereby isolating the influence of larval size composition on production performance.

An electric heater regulated at 30 °C was placed 1.2 m above the stacks. This configuration was retained as part of the evaluated production system and was not independently manipulated. Thus, any effect of tray position represents the combined influence of vertical location within the stack and the microenvironment generated by the heating and ventilation arrangement.

### 2.3. Diet and Feeding Schedule

Larvae were fed a feed mixture routinely used at the pilot production facility, consisting of commercial rabbit feed (NutriSow, GRAMOSA, Ezequiel Montes, Querétaro, Mexico), ground maize, and wheat bran. Within the feed mixture, these ingredients represented 30%, 20%, and 50%, respectively (Table 2). According to the manufacturer’s ingredient declaration, the commercial rabbit feed contained ground grains and cereal by-products, dehydrated alfalfa, oilseed meals and by-products, sugarcane molasses, vegetable oil, minerals, and a vitamin–mineral premix. The commercial rabbit feed was non-medicated and did not contain coccidiostats or exogenous enzymes. Batch-specific concentrations of individual amino acids and minerals, including methionine, lysine, calcium, and phosphorus, were not available and therefore could not be reported.

The three feed ingredients contained approximately 12% moisture (88% dry matter). The rearing substrate was prepared by adding 84 kg of water to 36 kg of the feed mixture, producing 120 kg of fresh substrate. Added water represented 70% of the fresh substrate mass but was not considered a feed ingredient in the calculation of dietary nutrient composition. Accounting for the intrinsic moisture of the feed ingredients, the freshly prepared substrate contained approximately 73.6% moisture and 26.4% dry matter. The rearing substrate was supplied at the beginning of the trial and again on days 8 and 15. At each feeding event, 6 kg of freshly prepared substrate was added to each tray. The substrate was placed at one end of the tray and larvae were placed at the opposite end.

The prepared rearing substrate contained 70% added water by fresh mass. Because the feed ingredients also contained intrinsic moisture, the calculated total moisture content of the freshly prepared substrate was 73.6%. This value refers to the substrate immediately after preparation and should be distinguished from the TDR-derived moisture measurements obtained during larval rearing, which are described below.

### 2.4. Environmental Monitoring

Substrate moisture was measured daily in all 40 experimental trays, with one CS616 time-domain reflectometry probe (TDR; Campbell Scientific, Logan, UT, USA) installed in each tray, and data were recorded with CR1000 dataloggers (Campbell Scientific, Logan, UT, USA). Each probe was initially positioned near the center of the tray, with its 30-cm sensing rods oriented longitudinally along the long axis of the tray. Probe depth was not mechanically fixed; therefore, changes in substrate depth and structure, together with larval activity, may have altered probe position during the rearing period. Substrate moisture was expressed as apparent volumetric water content (%, *v*/*v*).

Prior to deployment, the TDR probes were calibrated specifically for the experimental feed mixture by relating sensor readings to moisture content determined from dry matter measurements. However, calibration was performed without larvae and was not repeated during the rearing period. Because larval activity, feeding, and substrate consumption progressively altered substrate structure, bulk density, electrical conductivity, and depth, TDR values were interpreted primarily as relative indicators of temporal and positional changes in substrate hydric conditions rather than as exact measurements of absolute volumetric water content. Mean substrate moisture was initially evaluated as a covariate but was not retained in the final biomass model because it was not significant.

Substrate temperature was measured every three days using a single TCAV-L thermocouple probe (Campbell Scientific, Logan, UT, USA), which was sequentially rotated among the experimental trays. Measurements among trays were therefore not simultaneous. For each tray, measurements obtained throughout the 21-day rearing period were averaged and the resulting period mean was used as a covariate in the final biomass analysis. This approach was intended to characterize persistent differences in mean substrate temperature among experimental units rather than short-term thermal dynamics.

### 2.5. Harvest and Final Larval Biomass

After a rearing period of 21 days, live larvae were separated from the residual substrate (frass) using an automated rotary separator designed and constructed specifically for the production facility. This process allowed larval biomass and frass to be recovered as separate fractions. In this study, frass was operationally defined as the residual fraction recovered after mechanical separation of the larvae. No additional purification was performed to isolate larval excreta; therefore, this fraction could include larval excreta, residual or partially consumed feed, shed exuviae, and other substrate-derived material. Final larval counts were not recorded at harvest; therefore, survival could not be estimated. Consequently, final wet and dry larval biomass were interpreted as tray-level production responses integrating the combined effects of individual growth and survival.

After separation, representative fresh samples of approximately 400 g were collected from both the larval biomass and frass for dry matter determination. These samples were weighed, oven-dried at 80 °C until constant weight using a DKN900 drying oven (Yamato Scientific America Inc., Orangeburg, NY, USA), and weighed again to determine their dry matter content. Final dry larval biomass and dry frass production were calculated from the corresponding fresh recovered mass and dry matter fraction of the representative samples. Fresh and dried larval biomass and frass samples were weighed using a digital balance with a precision of ±0.1 g. Final dry larval biomass was considered the main productive response variable.

The remaining live larvae were subsequently exposed to 150 °C for 10 min in a custom-built rotary drying machine developed by the research team. This treatment was part of the routine processing protocol used at the pilot production facility. This high-temperature treatment was not used for analytical dry matter determination or for calculating final dry larval biomass. This thermal treatment simultaneously terminated the larvae and reduced their moisture content [29].

### 2.6. Statistical Analysis

The yield of black soldier fly was assessed in wet and dry basis to properly capture its production value across different applications. Wet weight reflects the actual harvest and is relevant for direct use in fresh feeding systems for certain animal species, where larvae are provided immediately after collection. However, because moisture content can vary widely, dry weight is essential to accurately quantify true biomass and nutrient yield, which are critical for processing into balanced animal feeds, such as meals or ingredients for aquaculture and livestock diets. The yield of frass was evaluated only on dry mass basis.

Response variables were analyzed using an analysis of covariance. Initial biological load and tray position within the stack were included as fixed effects. Stack was included as a blocking factor, and mean substrate temperature during the rearing period was included as a covariate. The statistical model was:

(1) $$Y_{ijk}=\mu +\alpha_{i}+\beta_{j}+\rho_{k}+\gamma x_{ijk}+\varepsilon_{ijk},$$

where $Y_{ijk}$ is the response variable, μ is the overall mean, $\alpha_{i}$ is the effect of the *i*th initial biological load level (i=1,…,5), $\beta_{j}$ is the effect of the *j*th tray position within the stack (j=1,…,5), $\rho_{k}$ is the effect of the *k*th stack (k=1,…,4), $x_{ijk}$ is the mean substrate temperature, γ is the regression coefficient associated with temperature, and $\varepsilon_{ijk}$ is the residual error. Dunnett’s test was used to compare treatment means with the control levels. The lowest level treatment (29 g tray^−1^) was used as the control for initial biological load, and the lowest tray position was used as the control for stack position. Statistical significance was evaluated at α=0.05.

Substrate moisture dynamics were analyzed with a mixed model for repeated observations through time. Initial biological load, tray position, time, and the interaction between initial biological load and tray position were included as fixed effects. A model with random slopes for time by stack was evaluated first, but the estimated random-slope variance was close to zero. Therefore, the final model retained stack as a random intercept:

(2) $$M_{ijkl}=\mu +\alpha_{i}+\beta_{j}+\tau_{l}+{\left(\alpha \beta \right)}_{ij}+u_{k}+\varepsilon_{ijkl},$$

where $M_{ijkl}$ is the substrate moisture observation, $\alpha_{i}$ is the effect of initial biological load, $\beta_{j}$ is the effect of tray position, $\tau_{l}$ is the effect of time, ${\left(\alpha \beta \right)}_{ij}$ is the interaction between initial biological load and tray position, $u_{k}$ is the random intercept for stack, and $\varepsilon_{ijkl}$ is the residual error. Model residuals were evaluated graphically. Normality was also assessed using the Anderson–Darling test, but graphical diagnostics were prioritized for judging model adequacy because the objective was to describe the main temporal and positional trends in substrate moisture. Statistical procedures were performed using SAS (version 9.4; SAS Institute Inc., Cary, NC, USA) and R (version 4.4.1; R Core Team, Viena, Austria).

## 3. Results

### 3.1. Final Larval Biomass as Affected by Initial Biological Load and Tray Position

The analysis focused on three response variables: final larval biomass on a wet basis, final larval biomass on a dry basis, and final frass production on a dry basis. The overall model was significant for all three responses. Initial biological load (α) was a significant source of variation for wet larval biomass, dry larval biomass, and dry frass production. Tray position within the stack (β) was significant for wet larval biomass and dry frass production, whereas its effect on dry larval biomass was not significant. Stack or block (ρ) was significant only for wet larval biomass. Mean substrate temperature during rearing (*x*) was significant for wet larval biomass, but it was not significant for dry larval biomass or dry frass production (Table 3).

### 3.2. Effect of Initial Biological Load

Dunnett–Hsu comparisons were used to compare each initial biological load level against the lowest initial biological load treatment (29 g tray^−1^). For wet larval biomass, only the 200 g tray^−1^ treatment differed from the control, showing a lower adjusted mean. For dry larval biomass, none of the initial biological load levels differed from the control after adjustment. For dry frass production, the 97 g tray^−1^ treatment produced more dry frass than the control, whereas the remaining treatments did not differ from the control (Table 4).

### 3.3. Effect of Tray Position Within the Stack

Tray position within the stack also modified production responses. For wet larval biomass, positions 3 and 4 produced significantly higher adjusted means than the bottom tray used as the control. Position 5 showed a numerically higher adjusted mean, but the Dunnett–Hsu-adjusted comparison did not reach the 0.05 significance level. For dry larval biomass, none of the tray positions differed from the bottom tray after adjustment. For dry frass production, only position 5 differed from the bottom tray, showing higher dry frass production (Table 5).

### 3.4. Temporal Dynamics of Substrate Moisture

**Figure 3:**
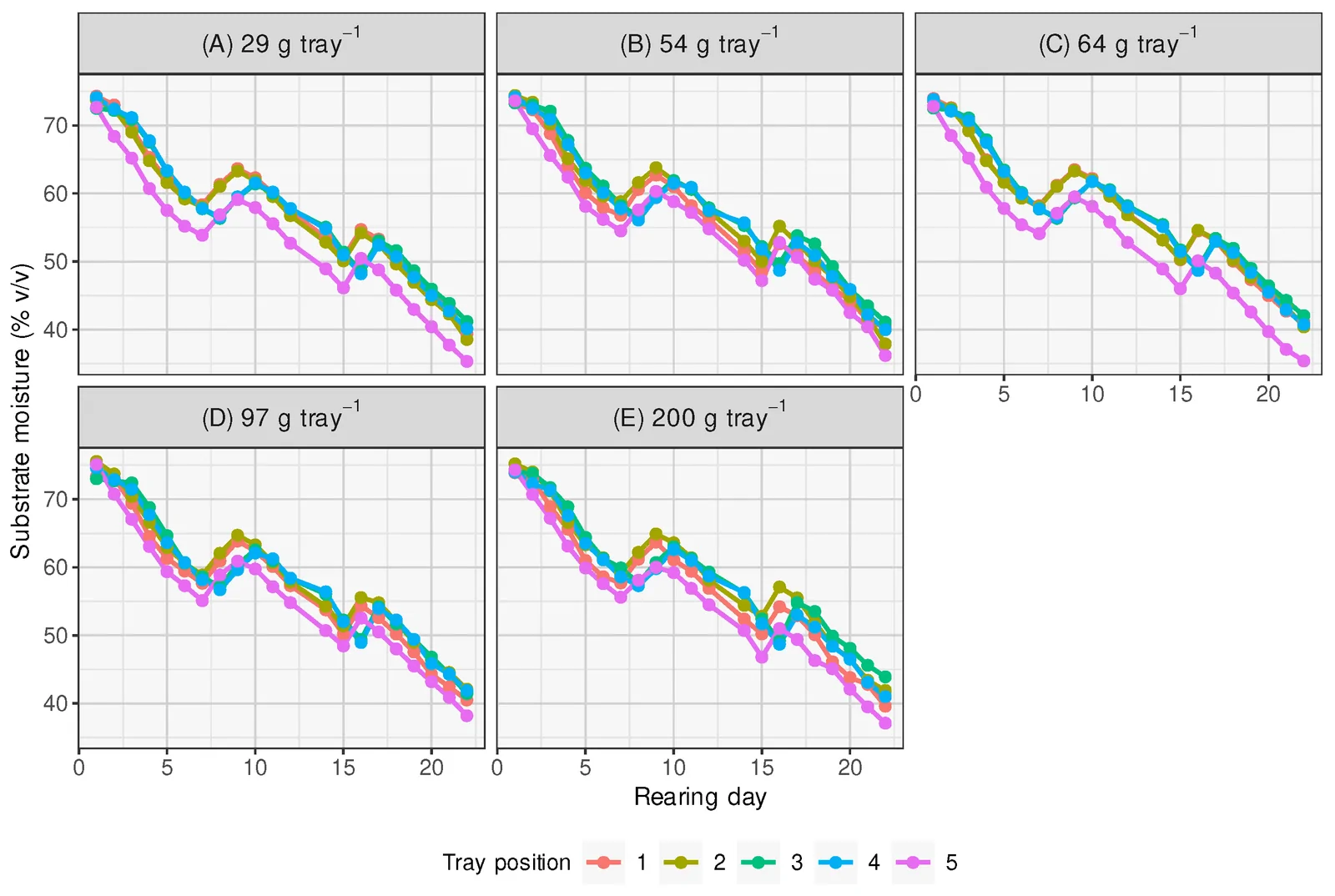
Temporal changes in TDR-derived substrate moisture according to tray position and initial biological load. Panels represent the five levels of initial biological load: (**A**) 29, (**B**) 54, (**C**) 64, (**D**) 97, and (**E**) 200 g tray^−1^. The sharp increases in substrate moisture on rearing days 8 and 15 correspond to scheduled re-feeding events, when 6 kg of freshly prepared rearing substrate was added to each tray. Lines and points represent mean substrate moisture across stacks. Tray 1 corresponds to the bottom position and Tray 5 to the top position within each stack.

Substrate moisture declined during the rearing cycle (Figure 3). A mixed model with random slopes for time by stack was evaluated, but the estimated variance for the random slope was close to zero. Therefore, the final model retained stack as a random intercept.

In the final mixed model, time had the strongest effect on substrate moisture (p<0.001), and tray position within the stack also affected moisture (p<0.001; Table 6). initial biological load and the interaction between initial biological load and tray position were not significant. These results show that moisture changed primarily as a function of elapsed rearing time and tray position, rather than as a direct response to the initial biological load treatment.

Residual diagnostics showed moderate deviations from normality in the tails, but the central region of the residual distribution was approximately symmetric. The model was therefore considered adequate to describe the main temporal and positional trends in substrate moisture.

### 3.5. Ancillary Analysis of Substrate Temperature

Because the heating equipment was located above the stacked trays, substrate temperature was examined to determine whether tray position produced a vertical thermal gradient. Mean substrate temperature was analyzed as an ancillary environmental response to determine whether the vertical position of the tray within the stack generated a thermal gradient. Mean substrate temperature did not differ significantly among tray positions (Table 7). In contrast, initial biological load affected mean substrate temperature (p<0.0001), with higher average temperatures observed in trays seeded with 97 and 200 g of larvae. Therefore, the observed effects of tray position on wet larval biomass and dry frass biomass were not accompanied by detectable differences in mean substrate temperature. No differences in period-mean substrate temperature were detected among tray positions. Under the intermittent and sequential measurement scheme used here, this result provides no evidence of a persistent large thermal difference among positions. However, short-lived temperature peaks or transient vertical gradients cannot be excluded because temperature was measured every three days and measurements among trays were not simultaneous.

### 3.6. Effect Size and Statistical Power Considerations

Effect-size estimates were used to complement the significance tests and to evaluate whether non-significant effects could still be relevant from a production perspective (Table 8). For wet larval biomass, initial biological load and tray position showed large effect sizes, with partial eta-squared values of 0.394 and 0.388, respectively. For dry larval biomass, initial biological load also showed a moderate-to-large effect size, whereas tray position showed a smaller but non-negligible effect size despite not reaching statistical significance. For dry frass biomass, initial biological load showed the largest effect size, followed by tray position.

The ancillary analysis of mean substrate temperature showed a contrasting pattern. initial biological load had a large effect on mean substrate temperature, whereas tray position had a negligible effect size. This result supports the interpretation that the vertical tray-position effect on production was not explained by persistent differences in mean temperature across the stack.

Because the design was unbalanced and included only 40 experimental units, the absence of statistical significance was interpreted together with the estimated magnitude of the effect. In particular, tray-position effects on dry larval biomass and some Dunnett–Hsu comparisons with borderline adjusted probabilities may represent biologically meaningful differences that the experiment had limited power to detect.

Power analysis should be treated prospectively. The present study provides estimates of residual variance and effect magnitude that can be used to design a balanced follow-up experiment. Rather than interpreting post-hoc power, future studies should define a minimum biologically important difference in final larval biomass, dry larval biomass, or frass production and then estimate the required number of trays per treatment to detect that difference with adequate power.

## 4. Discussion

### 4.1. Initial Biological Load and Tray-Level Biomass Recovery

This study evaluated how the initial biological load used to seed grow-out trays and the vertical position of the tray within a stack influenced the productive response of *Hermetia illucens*. Because all trays received the same number of larvae, the treatment should not be interpreted as numerical larval density. Instead, the treatment modified the initial biological load imposed on each tray through differences in individual larval mass. This distinction is important because a constant number of larvae can still generate different demands for oxygen, accessible substrate, moisture, and heat dissipation when larvae differ in size.

Final recovered larval biomass did not increase linearly with initial biological load. On a wet basis, the highest initial biological load produced the lowest adjusted final biomass and differed from the control. However, because survival and final individual larval mass were not quantified separately, the lower biomass recovery observed at the highest initial load cannot be attributed specifically to reduced individual growth, increased mortality, or a combination of both. The present results therefore demonstrate differences in tray-level biomass recovery among initial biological-load treatments rather than direct effects on individual larval growth. Previous work on black soldier fly production has shown that larval performance depends on the joint effect of density or biological load, feeding rate, moisture, temperature, and substrate physical properties rather than on a single factor acting alone [14,15,17,23,26,30,31]. Therefore, the lower performance observed at the greatest initial biological load is consistent with the idea that high biological load can intensify early competition, accelerate local substrate modification, and increase the metabolic demand of the tray before the system can sustain proportional growth.

This interpretation is also supported by the ancillary temperature analysis. Mean substrate temperature increased in trays seeded with greater initial biological load, even though tray position did not produce a vertical temperature gradient. Larval aggregation and biological activity are known to modify the thermal environment of the substrate, and this effect can either support assimilation or reduce performance when it alters aeration, moisture, or feed accessibility [20,31]. In the present study, temperature appeared to reflect biological load rather than stack position. Thus, temperature was not the main explanation for the tray-position effect, but it was a useful indicator that heavier initial larval loads changed the internal conditions of the rearing tray.

The dry-basis larval response showed a more conservative pattern: initial biological load affected the overall model, but Dunnett–Hsu comparisons did not detect differences from the control. This distinction between wet and dry biomass is biologically relevant. Wet biomass integrates both tissue accumulation and water content, whereas dry biomass better represents accumulated organic matter. The discrepancy suggests that treatments may have differed not only in larval growth, but also in larval water content, substrate moisture conditions, or the relative contribution of survival and size distribution to final biomass. For this reason, future experiments should include survival, individual final larval mass, size distribution, dry matter percentage, feed conversion, waste reduction, and frass recovery to separate growth from mortality and water-related changes in larval mass.

### 4.2. Frass Production and Resource Partitioning

Frass production responded differently from larval biomass. On a dry basis, frass was affected by initial biological load, and the 97 g treatment produced more frass than the control (29 g). This result suggests that the initial biological load influenced not only larval yield but also how the system partitioned substrate into residual material. In black soldier fly systems, frass is not simply an unused residue; it reflects the combined outcome of feed consumption, larval excretion, microbial transformation, substrate drying, and residual feed structure. Its production and composition are therefore strongly linked to the physical and biochemical dynamics of the substrate [9,10,11].

The fact that the highest initial biological load did not maximize larval biomass or frass production suggests that there was an operational threshold beyond which the system did not benefit from heavier larvae at seeding. A moderate initial biological load may have increased substrate turnover without creating the same level of constraint observed at the highest biological load. This finding is relevant for grow-out management because maximizing larval biomass and maximizing frass output may not occur at the same initial biological load. The preferred seeding strategy should therefore depend on the production objective: insect biomass, frass recovery, waste reduction, or a balance among these outputs.

### 4.3. Tray Position and Spatial Heterogeneity in Stacked Systems

Tray position within the stack was an important source of production heterogeneity. For wet larval biomass, intermediate positions produced higher adjusted means than the bottom tray, whereas for dry frass the top tray differed from the bottom tray. These results show that stacked systems should not be treated as collections of identical trays. Instead, each vertical position can represent a distinct microenvironment defined by airflow, moisture loss, gas exchange, access to heat, and the physical behavior of the substrate.

The ancillary temperature analysis clarified this point. Mean substrate temperature did not differ among tray positions, which indicates that the effect of tray position was not caused by a persistent vertical thermal gradient. This is important because the production system used an overhead heater; under that configuration, higher temperatures in upper trays would have been a plausible explanation. However, the adjusted temperature means were practically identical across tray positions. Therefore, the tray-position effect is more likely associated with moisture redistribution, ventilation, gas exchange, or localized substrate conditions than with average temperature. Although mean substrate temperature did not differ among tray positions, other unmeasured microenvironmental factors, such as localized airflow, moisture redistribution, or gas exchange, may have contributed to the position-specific responses. These mechanisms should be evaluated directly in future experiments.

This interpretation is consistent with evidence that moisture and substrate physical structure can strongly affect black soldier fly performance. Moisture influences larval mobility, ingestion, microbial activity, and oxygen availability, whereas substrate compaction and particle structure can alter aeration and access to feed [14,15,17,18,26]. In stacked trays, these processes may become spatially organized even when the same diet and the same number of larvae are used. The observed position effect therefore identifies stack architecture as a production factor, not merely as a space-saving arrangement.

The significant stack effect observed for wet larval biomass further supports the need to treat the physical production unit as part of the experimental design. Whole-stack conditions contributed to the final response, which means that future studies should include stack as a blocking factor or random effect. In industrial and pilot-scale systems, failure to account for stack and tray position can hide relevant sources of variability and reduce the repeatability of production estimates.

### 4.4. Moisture as the Dominant Process Variable

The combined results indicate that moisture was more closely aligned with the biological mechanism behind tray-position differences than temperature. Temperature differed among initial biological load levels, but not among tray positions. In contrast, the moisture analysis showed a strong temporal decline and a significant tray-position effect. Therefore, the productive differences among tray positions were more consistent with moisture- and ventilation-mediated constraints than with thermal stratification.

This distinction helps explain why mean substrate moisture was not retained as a direct predictor of final biomass in the final biomass model. Averaging moisture across the whole period can reduce a dynamic process into a single value and obscure biologically important events such as early drying, localized wet zones, or transient periods of poor aeration. Moisture may therefore act less as a static covariate and more as a process variable that changes the rearing environment through time. Previous studies have reported that black soldier fly larvae perform well within a broad but substrate-dependent moisture range, whereas suboptimal moisture can reduce growth, survival, and conversion efficiency [14,17,18,26,32]. Under the conditions of this experiment, moisture appears to have remained within a generally functional range, but its spatial and temporal variation likely contributed to the differences among tray positions.

The practical implication is that stacked rearing systems should monitor and manage moisture dynamically. A single initial diet moisture value is not enough to characterize the larval environment. Tray depth, side ventilation, larval movement, metabolic heat, and evaporative loss can cause moisture conditions to diverge among positions during the rearing cycle. For that reason, management strategies such as tray rotation, improved lateral ventilation, reduced stack height, or position-specific feeding and moisture correction should be evaluated in future trials.

### 4.5. Implications for System Design and Future Research

The main contribution of this study is the identification of two operational sources of variation in a stacked *H. illucens* rearing system: initial biological load and tray position. The results suggest that grow-out performance cannot be optimized only by selecting larger larvae before seeding. Larger larvae impose a greater biological load, and this load must match the capacity of the tray system to provide moisture, oxygen, feed access, and heat dissipation. Likewise, increasing the number of trays per stack may improve floor-space efficiency, but it can also increase production heterogeneity if moisture and ventilation are not homogeneous.

Future experiments should separate true numerical density from initial biological load. This would require factorial designs that independently vary larval number, individual larval mass, feeding rate, tray position, and airflow. Such designs would clarify whether the response is driven primarily by crowding, biological load, substrate availability, or microenvironmental limitation. In addition, future work should quantify oxygen, carbon dioxide, localized substrate temperature, moisture profiles, larval survival, individual larval size distribution, and frass quality. These measurements would allow stacked production systems to move from empirical management toward mechanistic control of rearing conditions.

Future experiments could use high-frequency TDR logging (e.g., 1–10 s intervals) to quantify short-term signal variability potentially associated with larval movement and to determine how stable the apparent moisture signal remains at different stages of substrate transformation. Also, the instrumentation should be modified to obtain paired and simultaneous time series for temperature and water content.

Overall, the findings support a management view in which *H. illucens* production is governed by the interaction between biological load and rearing-unit architecture. Under the conditions tested, the highest initial biological load did not maximize larval biomass, tray position generated production heterogeneity, and the absence of a positional temperature gradient points toward moisture and ventilation as the most likely drivers of the observed vertical effect. These results provide a basis for improving stacked tray systems through better control of moisture dynamics, airflow, and tray-position effects.

### 4.6. Effect Size, Power, and Biological Interpretation of Non-Significant Effects

The interpretation of the experiment should not rely only on statistical significance. The unbalanced design and the limited number of trays reduced the ability to detect some treatment differences, particularly for pairwise comparisons after Dunnett–Hsu adjustment. Therefore, effect-size estimates provide a more complete interpretation of the results.

Several effects that did not reach the conventional significance threshold still had non-trivial magnitudes. For example, tray position did not significantly affect dry larval biomass in the global test, but its effect-size estimate was not negligible. Similarly, some tray-position comparisons showed production differences that were large enough to be operationally relevant even when adjusted probabilities were slightly above 0.05. These results suggest that the experiment may have been underpowered for detecting some position-related differences in dry biomass.

In contrast, the absence of a tray-position effect on mean substrate temperature was supported by both the significance test and the effect-size estimate. The partial eta-squared value for tray position was close to zero, indicating that vertical position within the stack did not generate a meaningful thermal gradient when temperature was averaged across the rearing period. This distinction is important: non-significant effects with moderate effect sizes may indicate limited statistical power, whereas non-significant effects with negligible effect sizes provide stronger evidence that the factor had little practical influence.

From a production perspective, the most relevant non-significant results should therefore be treated as hypotheses for future experiments rather than dismissed. Future trials should use balanced replication and prospective power analysis based on the observed residual variance and the minimum biologically important difference in larval biomass or frass production. This approach would allow the experimental design to distinguish between statistically undetected but operationally relevant effects and truly negligible effects.

## 5. Conclusions

Initial biological load and tray position influenced production outcomes in the stacked *Hermetia illucens* rearing system evaluated in this study. Because all trays received the same number of larvae, the treatment represented initial biological load, not larval numerical density. Higher initial biological load did not produce proportional gains in final recovered larval biomass under the tested conditions. Because survival was not quantified, this production-level response cannot be partitioned into effects on individual growth and mortality.

Tray position contributed to production heterogeneity within the stack, particularly for wet larval biomass and dry frass output. However, the response did not follow a simple linear vertical gradient. The ancillary temperature analysis showed that mean substrate temperature did not differ among tray positions, suggesting that the position effect was not accompanied by detectable differences in period-mean substrate temperature; however, transient thermal gradients cannot be excluded by the present sampling design. Instead, the observed heterogeneity was more consistent with differences in moisture dynamics, airflow, gas exchange, or localized substrate conditions.

These results show that stacked tray systems should be optimized as integrated production units rather than as collections of equivalent trays. Future designs should evaluate airflow distribution, tray rotation, heater placement, and the maximum number of trays per stack to improve production uniformity. Follow-up experiments should use balanced replication and include survival, final individual larval mass, feed conversion, waste reduction, frass output, moisture dynamics, and gas exchange to identify the mechanisms driving the observed production differences.

From an operational perspective, trays within a stack should not be assumed to perform equivalently. Under the five-tray configuration evaluated here, the bottom position warrants particular attention to moisture and airflow management when uniform larval biomass production is the primary objective, because intermediate positions produced greater final wet biomass. The present experiment does not establish a maximum stack height; therefore, increases in the number of trays per stack should be validated by monitoring position-specific moisture conditions and production performance before implementation at commercial scale.

## Figures and Tables

**Table 1:** Initial biological load treatments established by varying the initial larval mass while maintaining a constant density of 9000 larvae per tray.

Initial Biological Load	Mean Individual Larval Mass	Biomass Density	Replicates
(g tray^−1^)	(g larva^−1^)	(g m^−2^)	(n)
29.25	0.003	157.7	11
53.64	0.006	289.1	5
63.90	0.007	344.5	9
96.84	0.011	522.0	10
200.16	0.022	1079.0	5

**Table 2:** Formulation and proximate composition of the feed mixture used to prepare the rearing substrate for *Hermetia illucens* larvae.

Feed Ingredient	Amount (kg) ^*a*^	Inclusion (%) ^*a*^	Proximate Composition ^*b*^		
			CP (%)	EE (%)	CF (%)
Commercial rabbit feed ^*c*^	10.8	30	15.5	5.0	14.0
Ground maize	7.2	20	8.8	3.8	2.0
Wheat bran	18.0	50	17.5	4.3	12.0
Total feed mixture	36.0	100	15.2	4.4	10.6

^*a*^ Amount and inclusion proportion of feed ingredients before water addition, on an as-fed basis. The three feed ingredients contained approximately 12% moisture (88% dry matter). ^*b*^ Proximate composition expressed on a dry-matter basis. CP = crude protein; EE = ether extract; CF = crude fiber. Total feed-mixture values were calculated from the ingredient inclusion proportions. ^*c*^ Commercial rabbit feed (NutriSow, GRAMOSA, Mexico). According to the manufacturer’s ingredient declaration, the feed contained ground grains and cereal by-products, dehydrated alfalfa, oilseed meals and by-products, sugarcane molasses, vegetable oil, minerals, and a vitamin–mineral premix. The product was non-medicated and did not contain coccidiostats or exogenous enzymes.

**Table 3:** Analysis of covariance for final larval biomass and frass production of *Hermetia illucens*.

Response	Source	df	SS	*F*	*p*
Wet larval biomass	Model	12	7.187	3.71	0.0023
	ρ	3	1.547	3.19	0.0394
	β	4	2.769	4.29	0.0082
	α	4	2.833	4.39	0.0073
	*x*	1	1.277	7.91	0.0091
	Error	27	4.361	–	–
Dry larval biomass	Model	12	1.330	2.73	0.0148
	ρ	3	0.083	0.68	0.5692
	β	4	0.348	2.14	0.1029
	α	4	0.517	3.18	0.0289
	*x*	1	0.128	3.15	0.0873
	Error	27	1.096	–	–
Dry frass production	Model	12	6.800	8.42	<0.0001
	ρ	3	0.455	2.25	0.1052
	β	4	0.744	2.76	0.0478
	α	4	1.462	5.43	0.0024
	*x*	1	0.083	1.23	0.2765
	Error	27	1.817	–	–

Note: α = initial biological load; β = tray position within the stack; ρ = stack/blocking factor; *x* = mean substrate temperature during rearing; df = degrees of freedom; SS = sum of squares. Wet larval biomass: $R^{2}=0.622$; dry larval biomass: $R^{2}=0.548$; dry frass production: $R^{2}=0.789$.

**Table 4:** Dunnett–Hsu-adjusted comparisons of final larval biomass and final dry frass production among initial biological load treatments.

	Final Wet Larval Biomass (kg)		Final Dry Larval Biomass (kg)		Final Dry Frass (kg)	
α (g tray^−1^)	LSM (SE)	p vs. C	LSM (SE)	p vs. C	LSM (SE)	p vs. C
29	3.542 (0.258)	C	0.729 (0.129)	C	1.998 (0.166)	C
54	3.304 (0.291)	0.9517	0.880 (0.146)	0.9017	2.092 (0.188)	0.9914
64	3.396 (0.312)	0.9917	0.588 (0.156)	0.9212	1.550 (0.201)	0.3987
97	3.025 (0.301)	0.2720	0.685 (0.151)	0.9930	2.740 (0.194)	0.0029
200	1.750 (0.407)	0.0250	0.167 (0.204)	0.2276	1.798 (0.263)	0.9531

Note: α = initial biological load; LSM = least-squares mean; SE = standard error; C = control treatment. The 29 g tray^−1^ treatment was used as the control for Dunnett–Hsu comparisons. Biomass and frass values correspond to final amounts recovered per experimental tray.

**Table 5:** Dunnett–Hsu-adjusted comparisons of final larval biomass and final dry frass production among tray positions within the stack.

	Final Wet Larval Biomass (kg)		Final Dry Larval Biomass (kg)		Final Dry Frass (kg)	
β	LSM (SE)	p vs. C	LSM (SE)	p vs. C	LSM (SE)	p vs. C
1	2.524 (0.178)	C	0.433 (0.089)	C	1.789 (0.115)	C
2	2.946 (0.151)	0.1585	0.573 (0.076)	0.4765	2.026 (0.098)	0.2539
3	3.142 (0.153)	0.0219	0.672 (0.077)	0.0955	2.021 (0.099)	0.2685
4	3.361 (0.151)	0.0016	0.667 (0.076)	0.1066	2.121 (0.098)	0.0659
5	3.046 (0.151)	0.0613	0.703 (0.076)	0.0517	2.221 (0.098)	0.0124

Note: β = tray position within the stack; LSM = least-squares mean; SE = standard error; C = control position. Position 1 corresponds to the bottom tray and was used as the control for Dunnett–Hsu comparisons. Biomass and frass values correspond to final amounts recovered per experimental tray.

**Table 6:** Mixed-model analysis of substrate moisture during larval rearing.

Source	Num. df	Den. df	SS	MS	*F*	*p*
α	4	2.84	149	37	4.5	0.130
β	4	806.26	1354	339	41.4	<0.001
τ	1	810.48	63,176	63,176	7724.1	<0.001
α×β	16	748.73	151	9	1.2	0.300

Note: α = initial biological load; β = tray position within the stack; τ = time; Num. df = numerator degrees of freedom; Den. df = denominator degrees of freedom; SS = sum of squares; MS = mean square. Stack was included as a random intercept to account for repeated observations through time.

**Table 7:** Ancillary analysis of mean substrate temperature during larval rearing.

Source	df	SS	MS	*F*	*p*
Model	8	105.47	13.18	10.55	<0.0001
β	4	0.14	0.04	0.03	0.9983
α	4	105.28	26.32	21.06	<0.0001
Error	31	38.75	1.25	–	–

Note: α = initial biological load; β = tray position within the stack; df = degrees of freedom; SS = sum of squares; MS = mean square. Temperature was averaged across the experimental period to reduce dimensionality and to evaluate whether tray position produced a persistent thermal gradient.

**Table 8:** Effect-size estimates for the main sources of variation in larval and frass outputs.

Response	Source	df	SS	$\eta_{p}^{2}$	$\omega_{p}^{2}$
Wet larval biomass	α	4	2.83	0.394	0.297
Wet larval biomass	β	4	2.77	0.388	0.288
Wet larval biomass	ρ	3	1.55	0.262	0.175
Wet larval biomass	*x*	1	1.28	0.227	0.192
Dry larval biomass	α	4	0.52	0.320	0.215
Dry larval biomass	β	4	0.35	0.241	0.125
Dry larval biomass	ρ	3	0.08	0.071	0.000
Dry larval biomass	*x*	1	0.13	0.104	0.069
Dry frass biomass	α	4	1.46	0.446	0.356
Dry frass biomass	β	4	0.74	0.290	0.181
Dry frass biomass	ρ	3	0.45	0.200	0.108
Dry frass biomass	*x*	1	0.08	0.044	0.008
Mean substrate temperature	α	4	105.28	0.731	0.690
Mean substrate temperature	β	4	0.14	0.004	0.000

Note: α = initial biological load; β = tray position within the stack; ρ = stack/blocking factor; *x* = mean substrate temperature during rearing. $\eta_{p}^{2}$ = partial eta squared; $\omega_{p}^{2}$ = partial omega squared. Negative $\omega_{p}^{2}$ estimates were truncated to zero.

## Data Availability

The raw data supporting the conclusions of this article will be made available by the authors on request.

## Abbreviations

The following abbreviations are used in this manuscript:

BSFL	Black soldier fly larvae
CF	Crude fiber
CP	Crude protein
CR1000	Campbell Scientific CR1000 datalogger
CS616	Campbell Scientific CS616 water content reflectometer
DM	Dry matter
EE	Ether extract
LSM	Least-squares mean
SE	Standard error
SS	Sum of squares
TDR	Time-domain reflectometry
